# Prevalence and impact of fertility concerns in young women with breast cancer

**DOI:** 10.1038/s41598-024-54961-6

**Published:** 2024-02-22

**Authors:** Samantha Mannion, Alexandra Higgins, Nicole Larson, Elizabeth A. Stewart, Zaraq Khan, Chandra Shenoy, Hazel B. Nichols, H. Irene Su, Ann H. Partridge, Charles L. Loprinzi, Fergus Couch, Janet E. Olson, Kathryn J. Ruddy

**Affiliations:** 1 Department of Medicine, Mayo Clinic, Rochester, MN USA; 2 Department of Oncology, Mayo Clinic, 200 1st St SW, Rochester, MN 55905 USA; 3 Department of Obstetrics and Gynecology, Mayo Clinic, Rochester, MN USA; 4 Department of Epidemiology, University of North Carolina, Chapel Hill, NC USA; 5 Department of Obstetrics and Gynecology, University of California San Diego, San Diego, CA USA; 6 Department of Medical Oncology, Dana-Farber Cancer Institute, Boston, MA USA; 7 Mayo Clinic Comprehensive Cancer Center, Mayo Clinic, Rochester, MN USA; 8 Department of Quantitative Health Sciences, Mayo Clinic, Rochester, MN USA

**Keywords:** Breast cancer, Quality of life, Cancer

## Abstract

Survey data from the Mayo Clinic Breast Disease Registry were used to assess fertility counseling and fertility preservation strategies in a modern cohort of young women with breast cancer. One hundred respondents were identified who were under age 50 at the time of breast cancer diagnosis and who expressed interest in future childbearing near the time of diagnosis and/or 1 year later. Ninety-three percent of the 81 respondents to the year one survey recalled fertility counseling prior to cancer treatment. Most who reported a high level of fertility concern declared that this concern had impacted their treatment decisions, often shortening their planned duration of endocrine therapy. Approximately half had taken steps to preserve future fertility, and a third had used a gonadotropin-releasing hormone agonist either alone or combined with another method (e.g., embryo or oocyte cryopreservation).

## Introduction

Breast cancer treatments can negatively impact fertility in several ways, including via the direct gonadotoxic effects of chemotherapy as well as via delays in childbearing due to adjuvant endocrine therapy^1^. Hormonally sensitive breast cancers are frequently treated with adjuvant anti-estrogen therapy for 5–10 years, during which time pregnancy is contraindicated and ovarian function may decline substantially^2^. The American Society of Clinical Oncology (ASCO) strongly recommends fertility counseling for all patients of reproductive age at the time of cancer diagnosis^3^. Standard fertility preservation techniques for women include oocyte and embryo cryopreservation. The 2018 ASCO Clinical Practice Guideline Update also recognized the conflicting evidence surrounding gonadotropin-releasing hormone (GnRH) agonists for fertility preservation and concluded that GnRH agonists may be offered to young women with breast cancer with the goal of reducing chemotherapy-induced ovarian insufficiency when other proven fertility preservation options are not available^3^.

Future fertility is an issue of concern for many young women with breast cancer; previous studies have estimated that about one-third of young women desire future children at the time of breast cancer diagnosis^4^, and half have some degree of concern about infertility following treatment^1^. Addressing this concern can decrease future regret and dissatisfaction concerning fertility^5^. However, reported rates of fertility counseling at diagnosis vary widely. One study of young women with breast cancer reported a counseling rate of only 26% as documented in the medical record between 2006 – 2014^6^, while another reported a rate of 68% based on patient recollection between 2006 – 2012^1^. In a 2008 survey of oncologists, 47% reported that they routinely referred patients for fertility preservation (all tumor types)^7^. A more recent 2015–2020 survey of Canadian surgical oncology practices and patients with breast cancer found an 84% counseling rate with a high degree of physician–patient concordance^8^. High rates of counseling have also been reported in settings with an oncofertility patient navigator (83%)^9^ and electronic health record-based prompting of fertility discussion (62%)^9,10^. However, some recent studies still identify suboptimal fertility counseling rates (12–41%)^11,12^.

Excellent safety data are available to allay concerns about the effect of fertility preservation and/or pregnancy on cancer-related outcomes, even in hormonally sensitive malignancies. Several studies have demonstrated that fertility preservation introduces no significant delay in time from diagnosis to treatment initiation in either the adjuvant or neo-adjuvant setting^13–15^. This is, in part, due to the introduction of random-start ovarian stimulation protocols that allow for expedited egg retrieval. Use of fertility preservation has not been shown to increase breast cancer recurrence risk, and aromatase inhibitor-based protocols can minimize hormonal surges during ovarian stimulation to alleviate any remaining concern^3,16–19^. Additionally, pregnancy after breast cancer is not associated with increased risk of cancer recurrence^19–22^.

The current study sought to investigate rates of fertility counseling, use of fertility preservation techniques, and how fertility concerns impact cancer treatment decisions in a contemporary cohort of patients with breast cancer seen at a large referral center in the United States.

## Methods

### Participant selection

Patients seen at least once at Mayo Clinic Rochester for a new diagnosis (within the year prior) of stage 0–4 breast cancer were invited (by mail or in person) to participate in the Mayo Clinic Breast Disease Registry (MCBDR). The patient population includes both local patients who received their initial diagnosis at Mayo Clinic as well as patients seeking a second opinion after initial diagnosis and/or treatment elsewhere. After informed consent, participants were asked to complete a baseline survey and annual follow-up surveys. Electronic charts were reviewed by a research nurse to collect information about tumor subtype, stage, and treatments received. The study cohort included women diagnosed with breast cancer under the age of 50 who answered either ‘yes’ or ‘unsure’ to the question: ‘At the present time, do you wish to have any/any more biological children in the future?’ on either the baseline or year 1 (Y1) survey (or both), with surveys completed between February 2015 and October 2020.

### Survey instruments and data analysis

Fertility concerns near the time of diagnosis were assessed on both surveys, with questions adapted from prior work by Ruddy et al.^1^. At baseline, women who indicated potential interest in future biological children were asked:

(1) How concerned are you about the possibility of not being able to become pregnant when you wish to? (Not at all concerned; a little concerned; somewhat concerned; very concerned).

On the Y1 survey, participants were asked to reflect on their feelings at the time of their diagnosis:

(2) When you were making your breast cancer treatment decisions, how concerned were you about the possibility of becoming infertile (unable to become pregnant) following your cancer treatment? (Not at all concerned; a little concerned; somewhat concerned; very concerned).

The Y1 survey also asked about impact of fertility concern on treatment decisions, receipt of fertility counseling and use of fertility preservation. These items included the following:

(3) When you were making your breast cancer treatment decisions, how much did your concern about becoming infertile (unable to become pregnant) following your cancer treatment impact on your decisions? (Not at all; A little; Somewhat; A lot).

(4) Have fertility concerns affected your treatment decisions in any of the following ways? (I have chosen not to take chemotherapy even though it was offered to me; I have chosen one chemotherapy regimen over another; I have chosen not to take tamoxifen or other hormonal medication [including ovarian suppression medication] even though it was offered to me; I have chosen or may choose to take tamoxifen or other hormonal medication for less than 5 years; Fertility concerns have not affected my treatment decisions; Other, please specify [with space for free text response]; None of the above).

(5) Prior to beginning therapy, did you discuss the issue of fertility (ability to become pregnant) following treatment with your doctors? (Yes; No).

(6) Before you began therapy or during therapy, did you take any special steps to lower the chance that you would become infertile with cancer treatment? (Yes; No).

(a) If yes, which steps did you take? (Cryopreservation [freezing] of embryos [fertilized eggs]; Cryopreservation [freezing] of eggs [unfertilized]; Cryopreservation [freezing] of ovarian tissue; GnRH agonist [e.g. Lupron or Zoladex shots]; Oral contraceptive pills [OCPs], Other, please specify [with space for free text response]; Not sure)

A physician (SM) performed a second chart abstraction to confirm stage and tumor biology data, purpose of GnRH agonist use (question 6), and to clarify free-text responses when needed. Responses of “other” for questions 4 and 6 were reclassified to one of the other options when possible; otherwise, these were left as “other.” Microsoft Excel was used to compute descriptive statistics from these data.

### Ethics declarations

The Mayo Clinic Breast Disease Registry was reviewed and approved by the Mayo Clinic Institutional Review Board (IRB 1815-04). Analysis of the data reported in this manuscript was reviewed and deemed exempt (IRB 21-005091). All participants gave informed consent prior to participation in the Mayo Clinic Breast Disease Registry. All research was carried out in compliance with relevant guidelines and regulations.

### Prior presentations

San Antonio Breast Cancer Symposium, December 2021: Mannion Samantha, Higgins Alexandra, Stewart Elizabeth A, Khan Zaraq, Shenoy Chandra, Larson Nicole, Nichols Hazel B, Su H Irene, Partridge Ann H, Couch Fergus J, Olson Janet E, Ruddy Kathryn. Prevalence and impact of fertility concerns in young women with breast cancer. San Antonio Breast Cancer Symposium. 2021; Abstract no. P4-11-02.

## Results

### Participant characteristics

**Figure 1:**
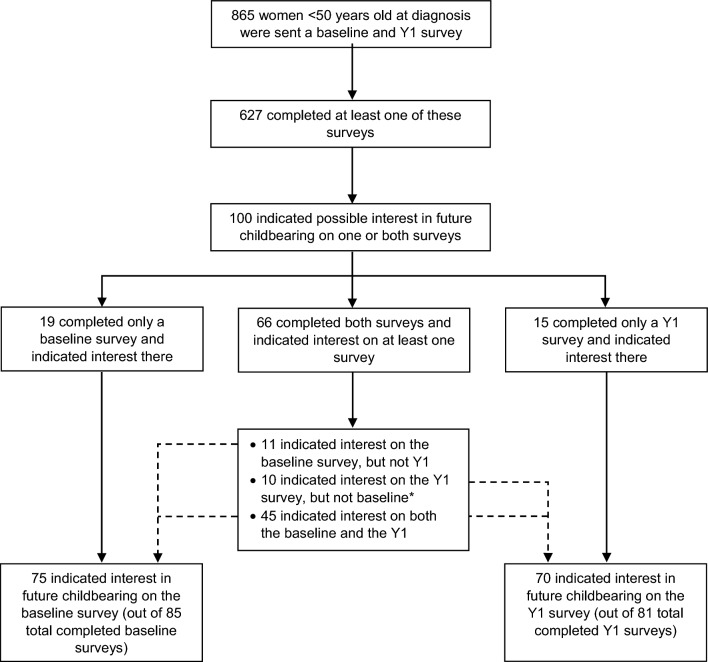
Participant selection. A participant was considered to have indicated interest if they answered, ‘yes’ or ‘unsure’ to the question: ‘At the present time, do you wish to have any/any more biological children in the future?’ *Of these 10, six selected ‘yes/unsure’ on the Y1 and ‘no’ on the baseline, and four selected ‘yes/unsure’ on the Y1 and left this question blank on the baseline.

A total of 865 women under age 50 at diagnosis were mailed a baseline and year 1 survey between February 2015 and October 2020 (Fig. 1). The registry received a completed baseline and/or Y1 survey from 627 patients, 100 of whom (16%) indicated interest in future biological children on one or both surveys (Table 1). Mean age at diagnosis was 33.8 years (standard deviation 5.2 years); the cohort included 90 women aged ≤ 40, 9 women aged 41–45, and 1 woman aged > 45 at diagnosis. The youngest member of the study population was 18 years old at diagnosis and the oldest was 47 years old.

**Table 1:** Characteristics of breast cancer survivors expressing possible desire for future biological children at baseline and/or year 1 (N = 100).

Participant characteristics	Number of participants
Age at cancer diagnosis, years	
18–40	90
41–45	9
> 45	1
Race	
White	92
Non-white or choose not to disclose	8
Ethnicity	
Not Hispanic or Latino	96
Hispanic or Latino or choose not to disclose	4
Nodal disease at diagnosis	
N0 or NX	72
N1–N3	28
Metastatic disease at diagnosis	
M0 or MX	98
M1	2
ER status	
ER positive	81
ER negative/missing	19
Chemotherapy	
Yes	68
No	32
Radiation therapy	
Yes	45
No/missing	55
Surgical status	
Mastectomy	78
Breast-conserving surgery	20
No surgery or missing	2
Endocrine therapy	
Yes	61
No/missing	39
Education	
High school degree and/or vocational education	5
Some college, associate degree, or bachelor’s degree	46
Graduate school	31
Missing	18
Marital status	
Married or living with partner	63
Separated, divorced, widowed, or never married	24
Missing	17
Menopausal status	
Periods have stopped	5
Periods have not stopped	59
Not sure/missing	36
Pregnancy history	
Previously pregnant	40
Pregnancy resulting in ≥ 1 live birth(s)	31
Never been pregnant/missing	60

For marital status, some respondents selected more than one option. Education history, marital status, menopausal status, and pregnancy history were self-reported on the baseline survey.

### Fertility concern and impact on treatment decisions

**Figure 2:**
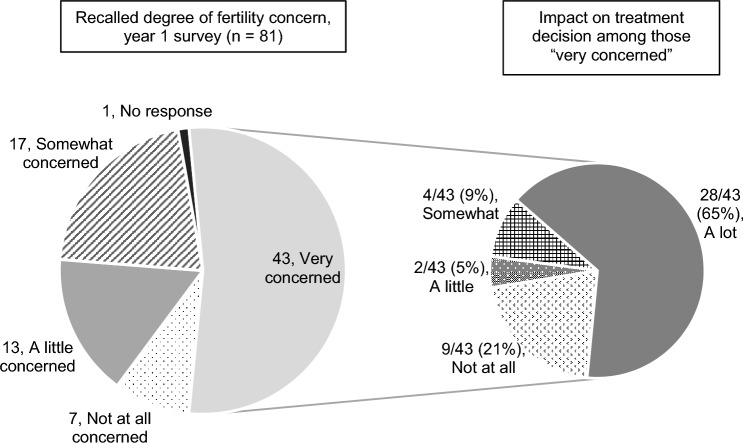
Degree of fertility concern and impact on treatment decision, year 1. The graph on the left demonstrates the degree of fertility concern recalled by respondents at the time of treatment decisions, as reported on the Y1 survey. The graph on the right shows data from a subset of respondents to display impact of fertility concern on treatment decision among those women who recalled that they were “very concerned”.

Of those who reported a possible or definite interest in future biological children on the baseline survey (n = 75), 28% concurrently reported that they felt ‘somewhat concerned’ and 40% ‘very concerned’ about the possibility of not becoming pregnant when they wished (question 1). Of those reporting a possible or definite interest in future biological children on the Y1 survey (n = 70), 17% concurrently reported that they recalled feeling ‘somewhat concerned’ and 59% ‘very concerned’ about the possibility of infertility when they were making their breast cancer treatment decisions (question 2). The majority (65%) of women who recalled on the Y1 survey feeing ‘very concerned’ about infertility when they were making their breast cancer treatment decisions (43 out of 81 total Y1 respondents) reported that this concern had impacted those decisions ‘a lot,’ which was the strongest available option on a 4-point Likert scale that included ‘not at all’, ‘a little’, ‘somewhat’ and ‘a lot’ (question 3) (Fig. 2). Of those who recalled on the Y1 survey feeling ‘somewhat concerned’ about infertility (n = 17), 59% reported ‘a little’ impact on treatment decision and 18% reported ‘somewhat’ of an impact.

**Figure 3:**
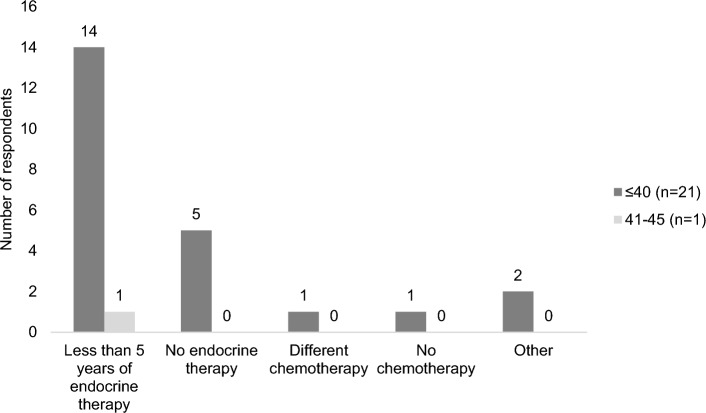
Treatment changes made in response to fertility concerns, year 1 (n = 22). Includes respondents who chose an answer to the question ‘Have fertility concerns affected your treatment decisions in any of the following ways?’ other than ‘no effect’ or ‘none of the above’ on the Y1 survey. Some respondents selected more than one answer. ‘Less endocrine therapy’ includes respondents who had chosen or may choose to take tamoxifen or other hormonal medication for less than 5 years. ‘No endocrine therapy’ includes respondents who had chosen not to take tamoxifen or other hormonal medication (including ovarian suppression medication) even though it was offered.

Participants were asked to indicate on the Y1 survey whether fertility concerns had impacted their treatment in several specific ways (question 4). Twenty-two indicated a treatment change (answers other than ‘no effect’ or ‘none of the above’). Most of these patients (21 of the 22) were aged 40 or younger and 1 was aged 41–45 at the time of diagnosis. The most common treatment change was a shortened duration of endocrine therapy, with 68% (15/22) indicating that they had chosen or may choose to take tamoxifen or other endocrine therapy for less than five years (Fig. 3). Five participants indicated that they had chosen to forego endocrine therapy entirely. Of these, two had ductal carcinoma in situ (DCIS) and chose bilateral mastectomy (perhaps to avoid endocrine therapy). Some respondents selected more than one type of treatment change.

There were six women who reported ‘no’ when queried about desire for future biological children at baseline who, at Y1, reported that they were ‘unsure’. Conversely, there were eleven women who reported possible desire for future biological children at baseline (four selected ‘yes’ and seven selected ‘not sure’) who reported ‘no’ desire for future biological children at Y1.

### Fertility preservation

**Figure 4:**
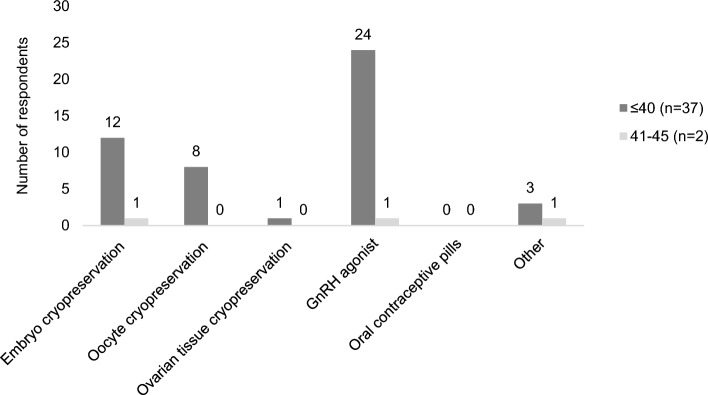
Methods of fertility preservation used (n = 39). Includes respondents who answered, ‘yes’ to the question ‘Before you began therapy or during therapy, did you take any special steps to lower the chance that you would become infertile with cancer treatment?’ on the Y1 survey. Some respondents selected more than one answer.

When queried on the Y1 survey, 93% of the 81 respondents recalled a discussion about fertility with their doctor prior to starting treatment (question 5). Before or during therapy, 48% of Y1 respondents (n = 39) had taken steps to lower the chance that they would become infertile with cancer treatment (question 6). Of these 39 women, 37 were aged 40 or younger and 2 were aged 41–45 at the time of diagnosis. Thirteen underwent embryo cryopreservation and eight underwent oocyte cryopreservation, representing 20 unique respondents (one reported both embryo and oocyte cryopreservation) (Fig. 4). Twenty-five had utilized a gonadotropin-releasing hormone (GnRH) agonist, and for 14, this was the only type of fertility preservation used. This represents a 31% (25/81) overall rate of GnRH agonist use among Y1 respondents. Chart review confirmed the accuracy of GnRH agonist use for the purpose of ovarian protection during chemotherapy in all cases. When examining only those Y1 respondents who received chemotherapy (n = 54), the rate of GnRH agonist use was 46% (25/54). Of the 39 participants who had utilized at least one fertility preservation strategy, 11 indicated that they had used more than one type. Most commonly, this was a combination of embryo cryopreservation and a GnRH agonist. Among Y1 respondents (n = 81), 47 were diagnosed prior to 2018 and 34 were diagnosed in 2018 or later. A higher rate of reported GnRH agonist use was observed in the among Y1 respondents diagnosed in 2018 or later (12/34, or 35%) compared to those diagnosed prior to 2018 (13/47, or 28%).

## Discussion

In this cohort of fertility-concerned survivors of young-onset breast cancer, about one-fourth used a gold-standard fertility preservation technique (e.g., oocyte or embryo cryopreservation; 20 of 81 Y1 respondents). This is similar to rates in the PREFER trial (18.2%)^23^ and in a study by Duraes et al. (2.4–17.1%)^24^, but higher than in some other published studies such as Swain et al. (3.3–4.2% among breast cancer patients)^11^ and Vu et al. (4–8% among all tumor types)^25^. The relatively high rate is likely due, in part, to the restriction of our sample to only those who self-identified an interest in future childbearing, instead of including all young breast cancer patients. The calculated overall uptake among the entire survey population (20/627; 3.2%) appears low, but must be interpreted with caution, as fertility preservation was only assessed in the subgroup with an interest in future childbearing (n = 81 Y1 respondents). Comparisons to other published studies are also limited by our inclusion of women up to age 49 (rather than only up to 40 or 45, like most other studies^11,23–25^).

Substantial GnRH agonist use was also observed, and slightly increased in women diagnosed after 2018. Recognition of the potential utility of GnRH agonists for fertility preservation by ASCO in the 2018 Clinical Practice Guideline Update was likely one factor leading to this increased use^3^. GnRH agonist administration was commonly combined with a gold-standard technique, but also reported in 14 cases to be the sole method of fertility preservation used. There are some data to suggest that GnRH agonists can help to preserve fertility by suppressing ovarian function^26,27^, with lower rates of primary ovarian insufficiency and higher rates of post-treatment pregnancy observed in a meta-analysis of patients treated with a GnRH agonist during chemotherapy^28^, though ASCO does not recommend substituting GnRH agonists for more established fertility preservation strategies^3^. A 31% rate of GnRH agonist use was observed in the current study (25 of 81 Y1 respondents), but this rate increased to 46% (25/54) when Y1 respondents who did not receive chemotherapy were excluded. Uptake of GnRH agonists varies widely in other published studies; the PREFER study of chemotherapy candidates found a very high rate of GnRH agonist use (> 90% among patients 18–45 years old)^23^. Two other studies found 24%^29^ and 3.1%^1^ rates of use when both chemotherapy recipients and non-recipients were included. Regional variability in the use of GnRH agonists may in part depend on cost/insurance coverage and whether a GnRH agonist is intended primarily to reduce the risk of infertility^1^ or also to reduce the risk of other sequalae of early menopause^23^. There is a strong recommendation in favor of GnRH agonist use for ovarian protection during chemotherapy in Italian guidelines^30^, where the PREFER study was conducted^23^. In our study, women with a high level of fertility concern often did report that this concern impacted their breast cancer treatment decisions, consistent with previous studies^31^.

This study identified a high rate (93%) of fertility counseling prior to treatment initiation. Notably, this was in a sample of women who had self-identified as being interested in future childbearing, which likely increased the rate of counseling compared to that in all young women, similar to in prior studies of fertility-concerned women^31^. Our survey did not ask who (patient or doctor) had initiated the fertility counseling discussion. When queried about specific types of treatment changes made in response to fertility concerns, patients most commonly identified a plan for a briefer duration of endocrine therapy. This is consistent with other literature identifying fertility concerns as a major factor in patients’ decisions to not initiate endocrine therapy or to discontinue it early^32,33^. The POSITIVE trial results support the safety of a two-year interruption of endocrine therapy to attempt pregnancy^34^, but this study did not assess early discontinuation or complete refusal of endocrine therapy or chemotherapy, all of which may increase risk of breast cancer recurrence.

Recall bias may impact our findings, as patients were asked on the Y1 survey to recall discussions of fertility preservation at the time of diagnosis. Additionally, the study population has limited racial diversity and over-representation of highly educated women, limiting generalizability of findings. While all participants were enrolled at a single site (Mayo Clinic Rochester), participants had often been diagnosed and/or received some of their cancer-directed therapy at other institutions.

We used a higher age cutoff (< 50 years old at the time of diagnosis) for our study than other published literature on this topic. This older age cutoff was selected to reflect increasing birthrates among women in their 40 s, which have more than doubled among women aged 40–44 in the United States from 1990 to 2019, from 5.57 per 1000 women to 12.96 per 1,000^35^. Similar trends are seen in Europe; the share of live births to mothers 40 or older in the European Union also increased between 2001 and 2021, from 2.4 to 5.7%^36^. This trend appears to be partially driven by utilization of assisted reproductive technology (ART) in this age group, with donor oocytes used by some women; births resulting from ART doubled in Denmark, Norway, and Sweden between 2008 and 2018^37^. Because older age at first birth is associated with a higher risk of breast cancer, it can be theorized that this trend may be even more prominent in women who are diagnosed with premenopausal breast cancer, leading to additional need for fertility preservation in this population. However, only two of the 39 women who pursued any fertility preservation technique were over the age of 40 at diagnosis, suggesting that uptake is still limited in older premenopausal patients. Fertility preservation should be routinely discussed with all pre-menopausal women at the time of diagnosis and clinicians should not make assumptions about interest in fertility preservation based on age.

In conclusion, this study provides updated insights about current rates of fertility counseling, how fertility concerns impact treatment decisions, and the frequency of use of specific of fertility preservation techniques. It is important to acknowledge the importance of this topic to young women with breast cancer and to err on the side of inclusivity when providing fertility counseling to facilitate reproductive options.

## Data Availability

The data underlying this article cannot be shared publicly to protect the privacy of individuals who participated in the study. The data will be shared on reasonable request to the corresponding author.
